# Prognostic value of KRAS codon 13 gene mutation for overall survival in colorectal cancer: Direct and indirect comparison meta-analysis

**DOI:** 10.1097/MD.0000000000007882

**Published:** 2017-09-01

**Authors:** Min Seob Kwak, Jae Myung Cha, Jin Young Yoon, Jung Won Jeon, Hyun Phil Shin, Hye Jung Chang, Hyung Kyung Kim, Kwang Ro Joo, Joung Il Lee

**Affiliations:** a Department of Internal Medicine; b Department of Pathology, Kyung Hee University Hospital at Gang Dong, Kyung Hee University College of Medicine, Seoul, Korea.

**Keywords:** codon 13 mutation, colorectal cancer, KRAS, meta-analysis, prognosis

## Abstract

**Background:**

The clinical significance of KRAS codon 13 mutation in patients with colorectal cancer (CRC) remains controversial. A systematic review and meta-analysis is necessary for a more precise estimation of the predictive role of KRAS codon 13 mutations in CRC patients.

**Methods:**

We performed a systematic search using the MEDLINE, EMBASE, and Cochrane library databases from January 2000 to November 2016. The prognostic value of KRAS codon 13 mutation for overall survival (OS) was investigated by measuring the hazard ratio (HR) and 95% confidence interval (CI). Data were analyzed with Review Manager Version 5.3 and the Canadian Agency for Drugs and Technologies in Health software.

**Results:**

OS in CRC patients with KRAS codon 13 mutation was worse than that in CRC patients with KRAS wild-type (pooled HR = 1.37, 95% CI: 1.03–1.81, *P* = .03). Subgroup analysis of studies of enrolled CRC patients treated with antiepidermal growth factor receptor (EGFR) therapy showed no significant difference in OS associated with KRAS codon 13 mutation in comparison to KRAS wild-type (pooled HR = 1.57, 95% CI: 0.98–2.51, *P* = .06). In the indirect comparison, no statistically significant association was observed between codon 12 and 13 mutations for OS in CRC patients (pooled HR = 0.88, 95% CI: 0.65–1.20, *P* = .43).

**Conclusion:**

The current meta-analysis suggests that Codon 13 mutation of KRAS gene seems to correlate with the OS of patients with CRC, but has similar OS to those with KRAS wild-type in patients receiving anti-EGFR therapy. No difference was detected in the OS of CRC patients with codon 13 mutation versus codon 12 mutation.

## Abstract 2

Supplemental Digital Content is available in the text

## 1 Introduction

Mutations of the KRAS gene, which are mainly localized in codons 12 and 13, have been found in up to 50% of cases of colorectal cancer (CRC).^[1]^ Until now, the prognostic significance of KRAS gene mutations has been unclear, as the results of previous studies are inconsistent.^[2–15]^ Some studies showed that KRAS gene mutations were associated with worse overall survival (OS) in patients with CRC,^[14,15]^ whereas other studies found no correlation.^[2,5]^ However, most previous studies included mutations for both codon 12 and codon 13 genes in their analysis of prognosis, without distinguishing between the mutations. In a few very recent studies, the effects of KRAS codon 13 gene mutations were analyzed separately from the effects of other codons.^[16–18]^ Recent accumulating evidence indicates that specific KRAS codon 12 and codon 13 gene mutations affect the functionality of KRAS proteins, thereby impacting clinical outcomes in CRC patients.^[14,19,20]^ Furthermore, KRAS codon 13 gene mutations have potential benefits in the context of anti-epidermal growth factor receptor (EGFR) drug therapy with better oncologic prognosis.^[14,20–27]^ However, the prognostic role of KRAS codon 13 mutations and prognostic differences between KRAS codon 12 and 13 mutations in CRC patients are unknown. To better understand this issue, a systematic review and meta-analysis of existing research is warranted.

The aim of this study was to assess the prognostic significance of KRAS codon 13 gene mutation in CRC patients.

## 2 Methods

### 2.1 Protocol and registration

We developed a protocol before conducting this review, but did not register it. The study was approved by the ethics committee of Kyung Hee University Hospital at Gang Dong.

### 2.2 Search strategy and selection criteria

We conducted a search of all available articles published from January 2000 to November 2016 in the PubMed (MEDLINE), EMBASE, and Cochrane library databases, using the terms “KRAS/KRAS gene/Genes, ras, G13D,” “mutations,” “mortality,” “survival/ prognosis,” and “colorectal cancer and/or adenocarcinoma or carcinoma or tumor or neoplasm.” Additional details on our search strategy are provided in Supplementary Table 1. The existing literature was reviewed for all observational studies and randomized controlled, retrospective/prospective cohort studies evaluating the significance between KRAS codon 13 gene mutation and OS in comparison to KRAS wild-type and/or other site mutations of the KRAS gene. The inclusion criteria and exclusion criteria were listed as follows. The first inclusion criteria was that the studies had the study outcomes about the predictive value of KRAS codon 13 gene mutation for OS in CRC patients and the provision of an estimated hazard ratio (HR) and its 95% confidence intervals (CI) of OS in the studies was the second inclusion criteria. We excluded studies if they included patients who received chemotherapy or radiation before surgery, or if they were non-human studies, published in English, conference abstracts, and repetition reports.

### 2.3 Data extraction

Two independent investigators (MSK and JMC) extracted specific data from each study, including author names, publication year, study location, total number of patients, number of cases according to specific KRAS gene mutational status, methods of mutation detection, HR with 95% CI of OS, and median follow-up duration. We assessed the risk of bias among primary studies. For methodological quality, the risk of bias in primary studies was assessed using criteria recommended by the Cochrane Collaboration for randomized studies^[28]^ and the Risk of Bias Assessment Tool for Nonrandomized Studies for nonrandomized studies.^[29]^ Disagreements were resolved through discussion.

### 2.4 Statistical analysis

The association between KRAS gene mutations and OS was measured by the pooled HR and 95% CI. Heterogeneity across studies was estimated using *I*^2^ statistics.^[30,31]^ If the results of heterogeneity tests were significant (*P* < .1 or *I*^2^ > 50%), then the random effects model was used to pool the estimate across studies with the DerSimonian-Laird method. Otherwise, the fixed-effects model was used.^[32,33]^ To investigate the possible sources of heterogeneity, we conducted subgroup analyses based on anti-EGFR therapy. An indirect treatment comparison between CRC patients with KRAS codon 12 mutations and codon 13 mutations was conducted with Canadian Agency for Drugs and Technologies in Health software using the Bucher method. The Bucher method is the simplest method to conduct indirect analysis (often referred to as a network meta-analysis).^[34,35]^

Given the small number of studies available, we were not able to perform meta-regression. Publication bias was assessed by graphical evaluation using only funnel plot asymmetry, and Egger's test was not conducted because of the limited power to detect small-study effects of publication bias.^[36,37]^ All statistical analyses including the risk of bias within studies and/or across studies were carried out with Review Manager Version 5.3 (RevMan; The Cochrane Collaboration 2014). The level of statistical significance was set at a *P* value < .05.

## 3 Results

### 3.1 Study selection and characteristics

**Figure 1:**
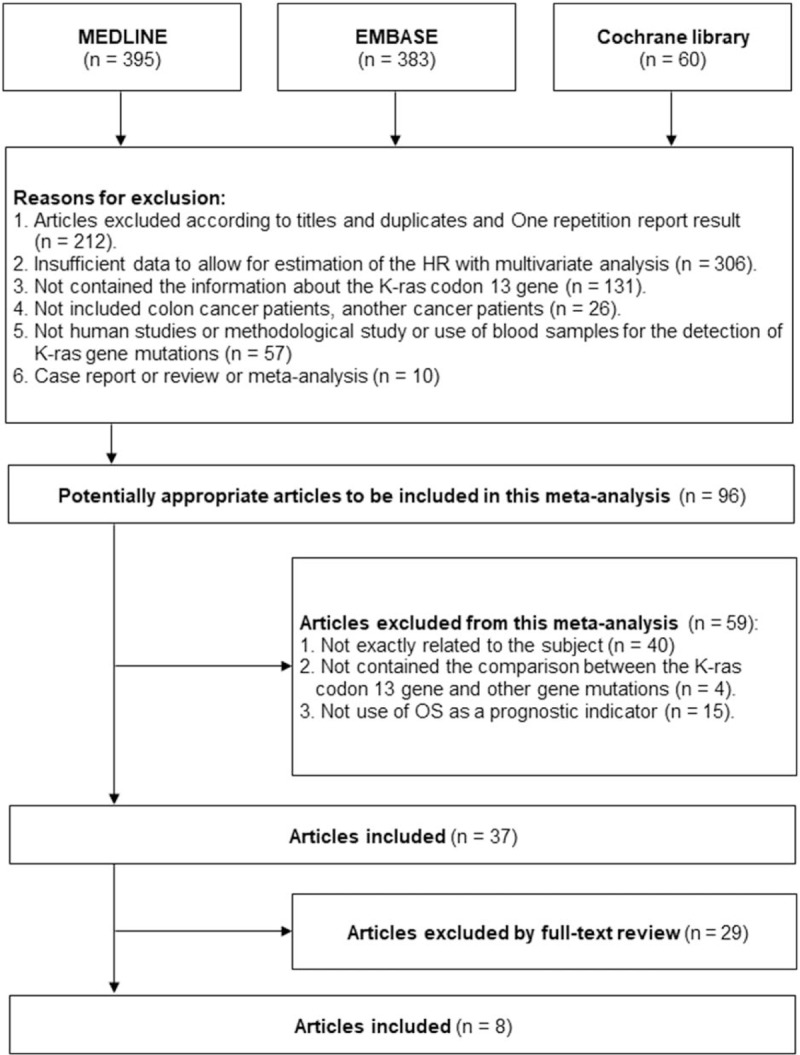
Flow chart of study identification and inclusion.

Our electronic search strategy identified 838 potentially relevant studies from designated databases (395 MEDLINE, 383 EMBASE, and 60 Cochrane library databases), of which 801 did not fulfill the inclusion criteria after careful examination of titles and abstracts. The remaining 37 articles were read in full and evaluated carefully by investigators. Finally, a total of 8 eligible published studies from China, Japan, Italy, the UK, Spain, the US, Sweden, and multinational origins^[14,17,23,38–43]^ were identified based on the inclusion and exclusion criteria. Of these, 2 studies by Tejpar et al^[23]^ and De Roock et al^[42]^ were carried out with datasets of previous clinical trials. In addition, the results from different patient groups according to treatment regimen were pooled independently. The detailed process of literature selection is shown in Fig. 1, and the main characteristics of each study are listed in Table 1. A total of 4223 patients with CRC were included after combining all studies. Among them, 1468 CRC patients (34.8%) had KRAS mutations, and 24.9% of the KRAS-mutated tumors had codon 13 gene mutations.

**Table 1:**
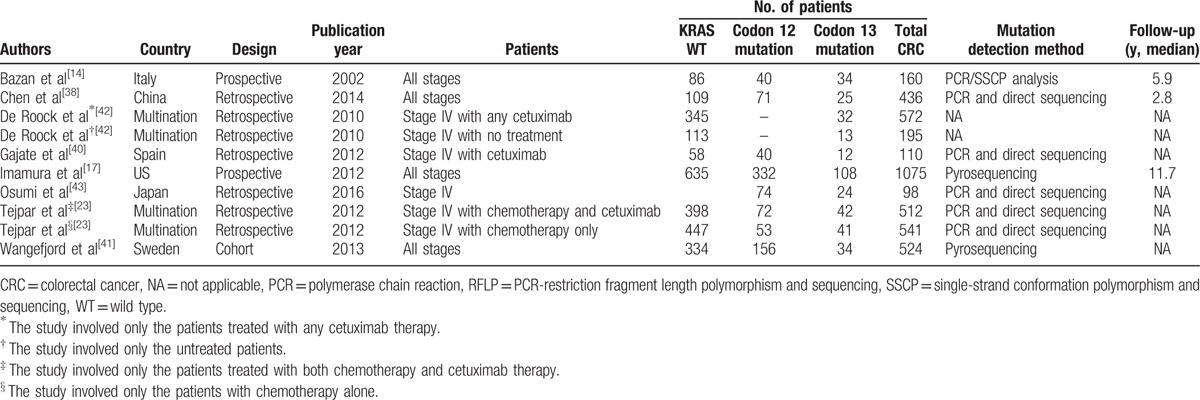
Characteristics of the included studies.

### 3.2 Quality assessment within studies

**Figure 2:**
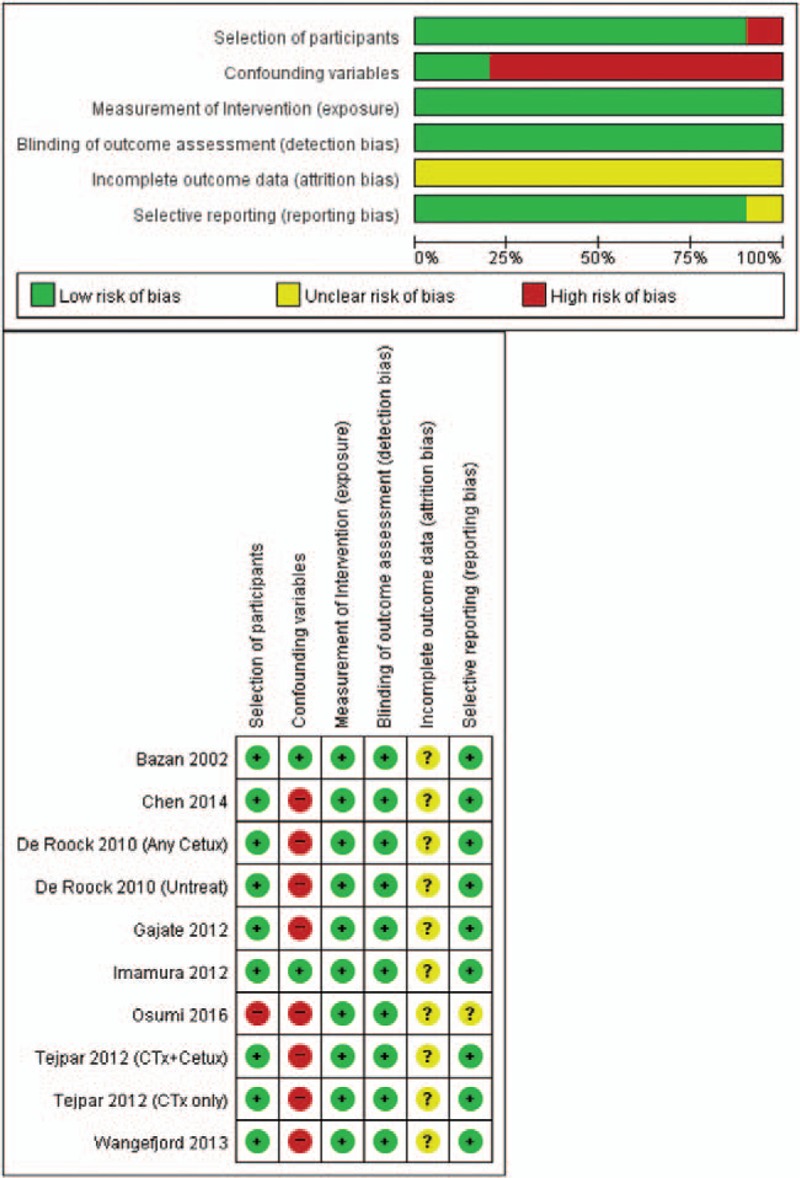
Risk of bias summary: review of author judgments about each risk of bias item for each included study based on the Risk of Bias Assessment Tool for Nonrandomized Studies (RoBANS) for observational studies. RoBANS = Risk of Bias Assessment Tool for Nonrandomized Studies.

Figure 2 presents the risk of bias assessment of nonrandomized studies with RoBANS. Because all 6 studies were either retrospective cohort or nonrandomized prospective studies, they were assessed as studies with low risk of selection, exposure, detection, and reporting. In all studies, the consideration of incomplete outcome data was not well described. The consideration of confounding variables contributed to our judgment of studies as having a high risk of bias.

### 3.3 Meta-analysis between KRAS wild-type and KRAS 13 gene mutations

**Figure 3:**
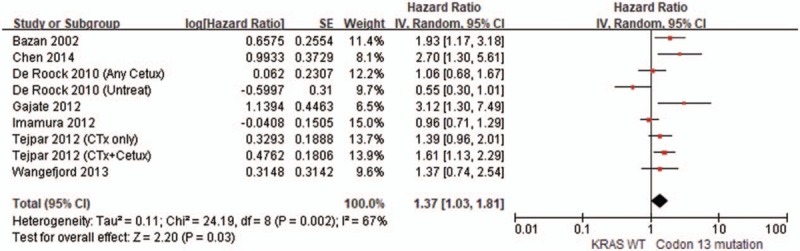
Forest plot of the comparison of codon 13 mutation vs KRAS WT in terms of overall survival.

**Figure 4:**
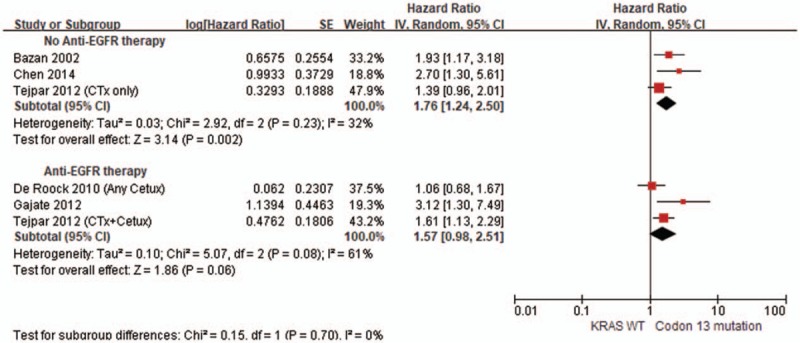
Forest plot of the comparison in subgroup analysis of codon 13 mutation vs KRAS WT in terms of overall survival.

As shown in Fig. 3, the pooled HR for the association between KRAS codon 13 gene mutations and OS in CRC patients was 1.37 (95% CI: 1.03–1.81, *P* = .03), with moderate heterogeneity between studies (*P* = .002, *I*^2^ = 67.0%). For further subgroup analysis based on the administration of anti-EGFR drugs, in studies of patients without anti-EGFR therapy, KRAS codon 13 gene mutation was associated with lower OS (pooled HR = 1.76, 95% CI: 1.24–2.50, *I*^2^ = 32.0%, *P* = .002) (Fig. 4). In contrast, there was no statistically significant association between KRAS codon 13 gene mutations and OS in studies of CRC patients with anti-EGFR therapy (pooled HR = 1.57, 95% CI: 0.98–2.51, *I*^2^ = 61.0%, *P* = .06) (Fig. 4).

### 3.4 Indirect comparison between KRAS codon 12 and codon 13 gene mutations

**Figure 5:**
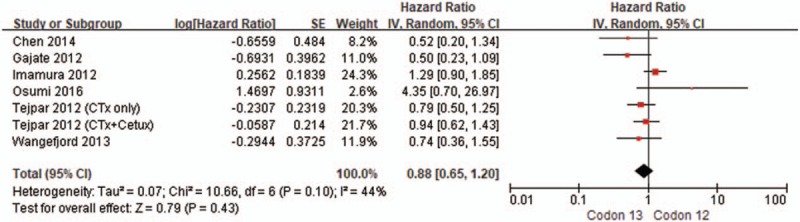
Forest plot of the indirect comparison of codon 13 mutation vs codon 12 mutation in terms of overall survival.

We identified 7 results of 6 studies^[17,23,38,40,41,43]^ that correspond to KRAS codon 12 and codon 13 gene mutations versus KRAS wild-type for OS in CRC patients and performed a direct head-to-head comparison of codon 12 and codon 13 mutations. For subsequent indirect comparison of codon mutations, we conducted a new meta-analysis to compare codon 12 versus codon 13 mutations. There was no statistically significant association between the 2 types of mutations for OS in patients with CRC (pooled HR = 0.88, 95% CI: 0.65–1.20, *I*^2^ = 44.0%, *P* = .43) (Fig. 5).

### 3.5 Publication bias

**Figure 6:**
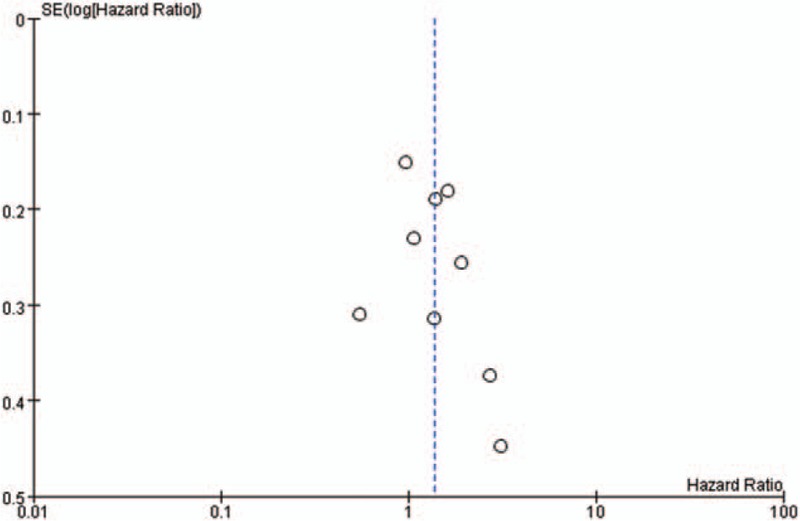
Funnel plot for publication bias of codon 13 for overall survival.

A funnel plot suggests minimal publication bias with central distribution of the included studies (Fig. 6).

## 4 Discussion

The results of this systematic review indicate that KRAS codon 13 gene mutation appears to have worse OS in comparison to KRAS wild-type in CRC patients, but shows similar OS in CRC patients with codon 12 gene mutation. Recently, a molecular epidemiological study by Imamura et al^[17]^ and computational analysis by Chen et al^[44]^ have shown that KRAS codon 13 gene mutations exhibit similar behavior to KRAS wild-type. A few subsequent clinical studies and meta-analysis have been conducted on CRC patients for this issue^[45–47]^; however, given the contradictory findings of the research, there is still no consensus. Our pooled results from the studies indicate a significant association with poorer OS for KRAS codon 13 gene mutation in comparison to KRAS wild-type (pooled HR = 1.37, 95% CI: 1.03–1.81, *I*^2^ = 67.0%, *P* = .03). Four sets of results among the enrolled studies showed a significantly unfavorable impact of KRAS codon 13 gene mutations on OS,^[14,38,40]^ whereas the remaining 5 sets of results failed to demonstrate any statistically significant association between KRAS codon 13 gene mutations and OS.^[17,39,41]^ Additionally, because anti-EGFR drug therapy potentially influences OS in CRC patients due to therapeutic effects, we conducted a subgroup analysis according to whether or not patients in the enrolled studies received anti-EGFR drug therapy. In the subgroup analysis, the studies of patients without anti-EGFR treatment showed that codon 13 gene mutation has a significantly worse OS in comparison to KRAS wild-type (pooled HR = 1.76, 95% CI: 1.24–2.50, *I*^2^ = 32.0%, *P* = .002). In contrast, the difference between codon 13 and KRAS wild-type in terms of OS in studies of CRC patients with anti-EGFR treatment were nonsignificant (pooled HR = 1.57, 95% CI: 0.98–2.51, *I*^2^ = 61.0%, *P* = .06). Several previous studies have indicated that codon 13 gene mutations in CRC patients may have different effects in the context of anti-EGFR therapy. In an in vitro study by Messner et al,^[48]^ anti-EGFR antibody treatment is shown to induce significant growth inhibition in tumor cell lines harboring a codon 13 mutation in contrast to cell lines with other KRAS mutations. A few clinical studies have also found that CRC patients with KRAS codon 13 mutations might benefit from anti-EGFR therapies.^[22,49]^ Our findings seem to support the potential benefits of anti-EGFR therapy for codon 13 mutations in CRC patients. Regardless, further studies are necessary because clinical applications of the data herein have not been fully clarified.

Another remarkable finding of this review is that indirect comparison meta-analyses demonstrate comparable OS in KRAS codon 13 and codon 12 gene mutations in patients with CRC. Whether or not KRAS codon 13 gene mutations can confer different CRC survival outcomes from other specific mutations—particularly codon 12 gene mutations—remains to be clarified. A previous in vitro study indicated that KRAS codon 13 gene mutations exhibit less potent tumorigenic activity than codon 12 mutations.^[50]^ Other experimental data have shown that KRAS codon 13-mutated tumors are less aggressive than codon 12-mutated tumors because they have higher levels of apoptosis.^[51]^ But, their mechanisms are still not fully understood. In contrast to these experimental studies, a clinical study by Modest et al^[52]^ showed that, in comparison to KRAS codon 12-mutated CRC, codon 13-mutated tumors have a more aggressive course, with local and distant metastatic disease presenting at the first diagnosis. These findings provide a rationale to investigate the potential differences of KRAS codon 13 gene mutation from KRAS codon 12 gene mutation, which may have a different prognostic value on OS in CRC patients. A recent study by Tejpar et al^[23]^ reports the first attempt to directly compare OS between KRAS codon 13 and codon 12 gene-mutated CRCs. However, this retrospective study was limited insofar as only metastatic CRC patients with chemotherapy-refractory disease were analyzed based on the small sample size. In present meta-analysis, including the results of the recent study,^[23]^ no significant difference in OS was observed between codon 13 and codon 12 mutations. The results of our indirect comparison meta-analysis showed a similar survival impact of both KRAS codon 13 and codon 12 gene mutations in CRC patients without statistical heterogeneity.

To the best of our knowledge, this is the first systematic review and meta-analysis to provide a head-to-head comparison of OS between KRAS codon 13 and codon 12 gene mutations using indirect comparison methods. Accordingly, it provides evidence-based information regarding differences between the 2 most frequently mutated sites of the KRAS gene. The findings, however, should be cautiously interpreted considering that the sample sizes of the included studies may be underpowered to detect differences in OS between the 2 codons.

There are several limitations of our meta-analysis. First, heterogeneity in the study populations is a potential problem. Colorectal cancer survival can be influenced by variable confounding factors, including geographic region, tumor stage, histologic type, or other underlying genetic mutations, such as P53^[53]^ and DCC.^[54]^ Second, the comparisons between KRAS codon 13 and codon 12 mutations were made indirectly using data generated from individual comparisons versus KRAS wild-type, because direct comparison data were not available. Therefore, direct comparative analysis on OS in CRC patients is warranted in terms of differences between the 2 codons. Third, insufficient information on age, sex, and other potential confounding factors may impact our results.

In conclusion, the current meta-analysis suggests that Codon 13 mutation of KRAS gene seems to correlate with the OS of patients with CRC, but has similar OS to those with KRAS wild-type in patients receiving anti-EGFR therapy. No difference was detected in the OS of CRC patients with codon 13 mutation versus codon 12 mutation. However, the relatively small sample size may not have enough statistical power to detect associations between the type of mutation and survival. Prospective investigations with larger sample sizes are required to validate this conclusion and to facilitate more accurate prediction of outcomes in patients with CRC.

## Supplementary Material

Supplemental Digital Content
