# Supplementary material for: Prognostic value of KRAS codon 13 gene mutation for overall survival in colorectal cancer: Direct and indirect comparison meta-analysis
Source: Medicine (Baltimore). 2017 Sep 1;96(35):e7882. doi: 10.1097/MD.0000000000007882 (PMC5585496; doi:10.1097/MD.0000000000007882)
Supplement: Supplemental Digital Content [file medi-96-e7882-s001.docx]

**Supplementary Table 1.** Search Strategy

**Study selection duration (publication year): 2000-1-1 ~ 2016-11-30**

**MEDLINE (Pubmed): 395**

1. (colon OR colorectal OR colonic OR rectal OR colorectum) AND (cancer OR cancers OR cancerous OR carcinoma* OR adenocarcinoma* OR neoplasm* OR neoplastic OR tumor OR tumors OR tumour* OR malignan* OR metastatic OR metastasis OR metastases)

2. outcome* OR ((treatment* OR chemotherap* OR protocol* OR regimen* OR drug OR drugs OR agent OR agents OR anti-neoplastic) AND (respond* OR response*)) OR failure* OR mortality OR fatal* OR death OR dead OR deaths OR "passed away" OR demise* OR Recurren* OR progression OR progressed OR relaps* OR growth OR grew OR growing OR remission* OR regression OR survival OR prognosis OR disease-free OR cure OR cures OR "Mortality"[Mesh] OR "mortality"[Subheading] OR "Survival"[Mesh] OR "Survival Analysis"[Mesh] OR "Neoplasm Recurrence, Local"[Mesh] OR "Neoplasm Recurrence, Locoregional"[Mesh]

3. K-ras OR KRAS OR "Genes, ras"[Mesh]

4. codon 13 OR "codon"[MeSH] OR G13D

5. 1 and 2 and 3 and 4

-------------------------------------------------------------------------------------------------------------

**Cochrane library: 60**

1. (colon OR colorectal OR colonic OR rectal OR colorectum) AND (cancer OR cancers OR cancerous OR carcinoma* OR adenocarcinoma* OR neoplasm* OR neoplastic OR tumor OR tumors OR tumour* OR malignan* OR metastatic OR metastasis OR metastases)

**and**

2. outcome* OR ((treatment* OR chemotherap* OR protocol* OR regimen* OR drug OR drugs OR agent OR agents OR anti-neoplastic) AND (respond* OR response*)) OR failure* OR mortality OR fatal* OR death OR dead OR deaths OR "passed away" OR demise* OR Recurren* OR progression OR progressed OR relaps* OR growth OR grew OR growing OR remission* OR regression OR survival OR prognosis OR disease-free OR cure OR cures

**Or using Mesh term as follows:**

3. "Mortality"[Mesh] OR "mortality"[Subheading] OR "Survival"[Mesh] OR "Survival Analysis"[Mesh] OR "Neoplasm Recurrence, Local"[Mesh] OR "Neoplasm Recurrence, Locoregional"[Mesh]

**and**

4. K-ras OR KRAS OR "Genes, ras"[Mesh]

**and**

5. codon 13 OR "codon"[MeSH] OR G13D

6. #1 and (#2 or #3 or #4 or #5 or #6 or #7 or #8) and (#9 or #10) and (#11 or #12)

-------------------------------------------------------------------------------------------------------------

**EMBASE: 383**

1. (colon OR colorectal OR colonic OR rectal OR colorectum) AND (cancer OR cancers OR cancerous OR carcinoma* OR adenocarcinoma* OR neoplasm* OR neoplastic OR tumor OR tumors OR tumour* OR malignan* OR metastatic OR metastasis)

2. outcome* OR ((treatment* OR chemotherap* OR protocol* OR regimen* OR drug OR drugs OR agent OR agents OR anti-neoplastic) AND (respond* OR response*)) OR failure* OR mortality OR fatal* OR death OR dead OR deaths OR "passed away" OR demise* OR Recurren* OR progression OR progressed OR relaps* OR 'cancer regression'/exp OR 'prognosis'/exp OR disease-free OR 'survival'/exp OR 'mortality'/exp

3. K-ras OR KRAS OR 'oncogene K ras'/exp

4. 'codon 13' OR G13D

5. 1 and 2 and 3 and 4
